# Rare Associations with Posterior Urethral Valves

**DOI:** 10.1155/2021/6647692

**Published:** 2021-04-26

**Authors:** Ahmed Osama Mohamed, Bala Eradi, Anthony Owen, Ashok Rajimwale

**Affiliations:** Department of Paediatric Surgery, Leicester Royal Infirmary, Infirmary Square, leicester, LE1 5WW, UK

## Abstract

Posterior urethral valves are a common cause of congenital bladder outlet obstruction. Known associations include cardiac malformations and gastrointestinal abnormalities. In this case series, we report on two cases of PUV associated with anorectal malformations along with a case of PUV in monochorionic diamniotic twins. We explore the difficulty in achieving a diagnosis and the final management. The association of posterior urethral valves in a patient with anorectal malformation should be suspected in case of associated oligohydramnios or oliguria postnatally. There should be a high index of suspicion in twin pregnancy even if only one of the twins is suspected of bladder outlet obstruction.

## 1. Introduction

Posterior urethral valves (PUV) are the most common congenital cause of bladder outlet obstruction in children and a leading cause of end-stage renal failure. The estimated incidence of PUV is 1 in 3800 live births in the UK, of which 40% of patients are diagnosed antenatally [1]. Nonurological common anomalies associated with PUV include cardiac malformations, gastrointestinal abnormalities, and aneuploidy in about 40% of cases [2]. We describe 3 cases of PUV with rare associations and their management.

## 2. Case 1: PUV Associated with Imperforate Anus

A baby boy born at 34 + 6 weeks by emergency C-section weighing 2.735 kg with antenatal scans suggestive of oligohydramnios and dilated bowel loops however with a normal renal tract. At birth, he was found to have an imperforate anus; hence, a sigmoid colostomy was performed.

Postoperatively, he was noticed to have oliguria and worsening renal functions. Interestingly at the same time, the colostomy bag was filling with faeces and watery effluent. Close inspection revealed watery discharge from defunctioning limb of colostomy explaining rising blood urea and creatinine levels and acidosis. We suspected bladder outflow obstruction and regurgitation of urine through the recto urethral fistula. Renal ultrasound scan (USS) showed bilateral hydronephrosis and hydroureters. Perineal USS showed dilated posterior urethra. Attempted urethral catheterisation proved difficult, so a suprapubic catheter was inserted.

**Figure 1:**
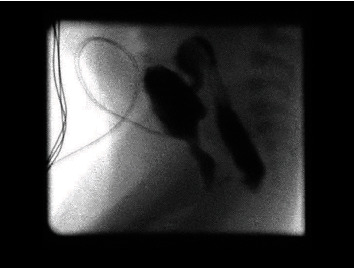
MCUG showing evidence of PUV and rectobulbar fistula.

Micturating cystourethrogram (MCUG) through suprapubic catheter revealed a dilated posterior urethra and a rectobulbar fistula (Figure 1). On day 13 of life and after stabilisation of general condition, the patient was taken to theatre for cystoscopic ablation of the valves. The suprapubic catheter was removed, and a urinary catheter was left for one week.

Subsequently, he suffered from persistent nonbilious vomiting along with metabolic alkalosis. Abdominal USS revealed coexisting pyloric stenosis. Laparoscopic pyloromyotomy was performed and made good postoperative recovery. At the age of 4 months, posterior sagittal anorectoplasty (PSARP) with dissection and ligation of the rectobulbar fistula was done. Under the same anaesthetic, repeat cystoscopy was done to confirm the absence of any residual valves. After regular dilatation and achieving a good size neo-anus, stoma closure was performed at the age of 7 months. On follow-up at 3 years, he is thriving well with improvement of the bilateral hydronephrosis and hydroureter on serial ultrasound scans and no history of urinary tract infections (UTI). He has achieved bladder control and has been progressing well with bowel continence.

## 3. Case 2: PUV Associated with Rectoperineal Fistula

**Figure 2:**
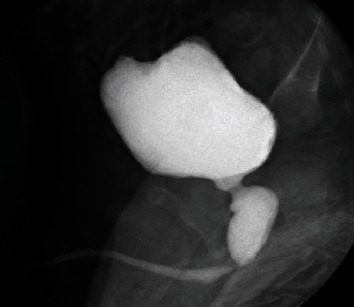
Dilated posterior urethra on MCUG.

This full-term baby boy was born after an uneventful pregnancy and normal antenatal scans. At birth, he was found to have a rectoperineal fistula, accessory nipples, and preauricular skin tags. He underwent primary PSARP on day two of life for the rectoperineal fistula. Echocardiography revealed a patent foramen ovale and tricuspid valve thickening. The rest of his VACTERYL screening—including a renal ultrasound—were normal. Unfortunately, this child later presented with two confirmed episodes of E-coli urinary tract infections in the first 4 months of life. Although a repeat USS did not reveal any anomalies, we proceeded for a MCUG, which showed a dilated posterior urethra suggestive of PUV (Figure 2). Cystoscopic valve ablation was then performed with good postoperative recovery. At the age of 8 years, this child has done well with normal renal function and has achieved both urinary and faecal continence with the aid of regular laxatives.

## 4. Cases 3 and 4: PUV in Monochorionic Twins

We report a case of PUV in monochorionic diamniotic twins born at 36 weeks by Emergency C-section due to breech presentation. Antenatal scans in twin 1 showed bilateral hydroureter and hydronephrosis and oligohydramnios. Postnatal renal USS showed bilateral hydronephrosis with loss of corticomedullary differentiation and a dilated left ureter.

**Figure 3:**
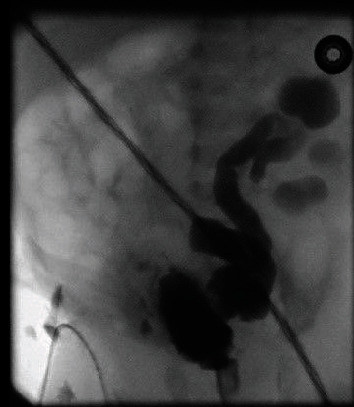
Dilated posterior urethra and left grade V reflux.

**Figure 4:**
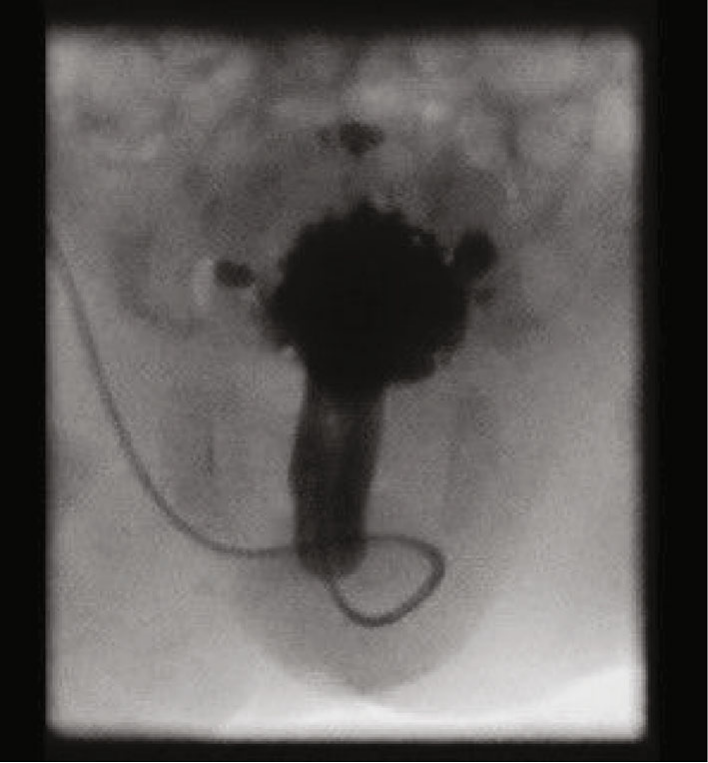
Dilated posterior urethra and a trabeculated urinary bladder.

Antenatal scan in twin 2 showed left hydronephrosis. Postnatal renal USS showed thick wall bladder and bilateral hydronephrosis and hydroureters. MCUG in twin 2 revealed a dilated posterior urethra and grade V left ureteric reflux (Figure 3) While in twin 1 revealed a grossly dilated posterior urethra and a trabeculated urinary bladder (Figure 4). Bladder drainage was achieved using urethral catheters in both twins. Due to the low birth weights of twins and circumstances related to the COVID-19 Pandemic, ablation was performed at 8 weeks of age. Currently, twins are doing well and awaiting check cystoscopy (accepted unit policy in our hospital) and circumcision.

## 5. Discussion

Posterior urethral valves (PUV) are thought to result from an anomalous insertion of the mesonephric duct into the urogenital sinus, preventing normal migration of these ducts and their anterior fusion forming the abnormal ridges in the membranous urethra [ 3]. Young's [4] classification of 3 types of PUV has recently been challenged, and a newer concept was proposed by Dewan and Goh that in bladder outlet obstruction, the uninstrumented urethra looks more like a circumferential obstructing membrane with a small central or eccentric opening named congenital obstructing posterior urethral membrane (COPUM) [5]. Various associations (up to 40%) with PUV have been reported including cardiovascular abnormalities, absent external auditory meatus, bilateral adrenal agenesis, hypospadias, micro/macrophallus, and anterior urethral valves [6–8].

The association of PUV with anorectal malformations is exceedingly rare and can pose a diagnostic and therapeutic challenge. Few reports [6, 9, 10] exist of this association; however, only one report had a presentation similar to our first case explaining the dilemma and difficulty identifying or suspecting PUV with anorectal malformations. Case 2 highlights the false-negative diagnosis of PUV on antenatal scans and normal postnatal scans (as a normal VACTREL screening tool in our institution); however, a diagnosis was made following investigation for recurrent UTI.

Comparing both cases, the presence of a rectourethral fistula in the first case could have served as a “pop-off” mechanism and protected the upper urinary tracts. This is evident when comparing the degree of posterior urethral dilatation in both cases.

Familial occurrence of PUV has been reported, particularly in dizygotic twins; however, P. Burttet in an extensive review of literature found 12 cases of familial nontwin sibling with PUV [11]. Interestingly, he also found that diagnosis in the second sibling was delayed in most cases if not suspected antenatally. It has been a known fact that the prevalence of structural defects is 1.2–2 times higher twin pregnancies compared to singletons, with increased rates in monochorionic twins [12]. This is also the case with PUV whose association with twins has been previously reported [13–15]. In one report, antenatal presentation in the identical twins was different in both including one twin showing urinary ascites and bilateral dysplastic kidneys while the other twin presenting a less severe form of the disease [15]. However, our twins had shown almost identical antenatal image of bilateral hydroureter and hydronephrosis though oligohydramnios was present in twin 1. Postnatal MCUG revealed the presence of reflux in one of the twins only. Although there is no established genetic link to this anomaly, the association of PUV in twins and nontwin siblings has led to the speculation of a possibility of an autosomal recessive pattern of inheritance or some form of genetic predisposition [14, 16]. This has led to the general recommendation of urologic evaluation of the male sibling and family member of a male child with PUV [13, 17].

## 6. Conclusion

Although exceedingly rare, the association of posterior urethral valves in a patient with anorectal malformation (ARM) should be suspected in case of associated oligohydramnios, postnatal oliguria, or watery effluents in colostomy bag with rising renal function and acidosis. There should also be a high index of suspicion in twin pregnancy even if only one of the twins is suspected of bladder outlet obstruction.

## Data Availability

The data used to support the findings of this study are included within the article.
